# Plumbagin Prevents Secretory Diarrhea by Inhibiting CaCC and CFTR Channel Activities

**DOI:** 10.3389/fphar.2019.01181

**Published:** 2019-10-09

**Authors:** Bo Yu, Xiaojuan Zhu, Xinyu Yang, Lingling Jin, Jia Xu, Tonghui Ma, Hong Yang

**Affiliations:** ^1^School of Life Sciences, Liaoning Provincial Key Laboratory of Biotechnology and Drug Discovery, Liaoning Normal University, Dalian, China; ^2^College of Basic Medical Sciences, Dalian Medical University, Dalian, China

**Keywords:** plumbagin, secretory diarrhea, cystic fibrosis transmembrane conductance regulator, calcium-activated chloride channel, intestinal motility

## Abstract

Secretory diarrhea, which primarily originates through intestinal pathogens and viruses, is a health burden in many regions worldwide. Enterocyte Cl^−^ channels, as the final step in enterotoxin-induced fluid secretion, constitute an attractive class of targets for diarrhea therapy. Chloride channel inhibitors have become a new class of candidates for antisecretion and anti-intestinal motility agents. In the present study, we identified plumbagin as a *transmembrane protein 16A* (*TMEM16A*) inhibitor in a cell-based fluorescence-quenching assay, and the IC_50_ value was ∼12.46 µM. Short-circuit current measurements showed that plumbagin reversibly inhibited the E_act_-induced Cl^−^ current on the apical side of *TMEM16A*-transfected Fischer rat thyroid (FRT) cells with no significant effect on cytoplasmic Ca^2+^ signaling. Notably, plumbagin also inhibited the activity of intestinal epithelial calcium-activated chloride channel (CaCC) and cystic fibrosis transmembrane conductance regulator (CFTR) in both HT-29 cells and mouse colons, but had no effects on the activity of the Na^+^-K^+^ ATPase or K^+^ channels. In *in vivo* experiments, the administration of plumbagin reduced both *Escherichia coli* heat-stable enterotoxin (STa)- and cholera toxin (CT)-induced intestinal fluid secretion. In neonatal mouse models of CT- and rotavirus infection-induced diarrhea, 0.4 µg plumbagin inhibited secretory diarrhea by >40% and 50%, respectively, without affecting intestinal epithelial integrity or the rotaviral infection. In addition, plumbagin exerted inhibitory effects on the vasoactive intestinal peptide (VIP)-, prostaglandin E2 (PGE2)-, and 5-hydroxytryptamine (5-HT)-stimulated Cl^−^ currents. In the evaluations of intestinal motility, plumbagin significantly delayed intestinal motility and inhibited intestinal smooth muscle contractility without an evident impact on contractive frequency. Collectively, our results indicate that plumbagin inhibits both Ca^2+^- and cAMP-activated Cl^−^ channels, accounting for the mechanisms of plumbagin inhibition of chloride secretion and intestinal motility. Thus, plumbagin can be a lead compound in the treatment of CT-induced, Traveler’s, and rotaviral diarrhea, as well as other types of secretory diarrhea that result from excessive intestinal fluid secretion and increased intestinal peristalsis.

## Introduction

Secretory diarrhea has a severe impact on both the mortality and morbidity of patients in all age groups and from different geographical locations, and this malady remains a leading cause of death in children under 5 years old worldwide (Walker et al., 2013). Secretory diarrhea is the result of increased intestinal fluid secretion, which is mainly due to chloride secretion from the intestinal epithelial cells in the lumen and future water exudation along the paracellular spaces (Schiller, 1999).

The main chloride channels involved in intestinal fluid secretion are cystic fibrosis transmembrane conductance regulator (CFTR) and calcium-activated chloride channel (CaCC) (Barrett and Keely, 2000). CFTR is a cAMP-dependent chloride channel that is expressed in the epithelial cells of all tissues related to fluid secretion and absorption (Kunzelmann, 1999). In the intestine, the excessive stimulation of CFTR causes the excessive release of intestinal liquid and stool output (Moon et al., 2015; Cil et al., 2016). Compared to that of CFTR, the contribution of CaCC to intestinal fluid secretion is still unclear. *Transmembrane protein 16A* (*TMEM16A*) was the ﬁrst CaCC protein to be identified and was once thought to be a candidate intestinal epithelial CaCC (Ousingsawat et al., 2009); however, some researchers believe that a non-*TMEM16A* CaCC exists in the intestinal epithelium (Namkung et al., 2011). On the other hand, there is increasing evidence that *TMEM16A* plays an important role in the regulation of intestinal motility (Sanders et al., 2012), which is based on the following results: (a) the interstitial cells of Cajal (ICC) highly express *TMEM16A* (Isozaki et al., 1997; He et al., 2001); (b) the major function of the ICC is to participate in intestinal electric slow-wave generation and transmission and the regulation of intestinal smooth muscle contractions (Sanders and Ward, 2006); (c) pharmacological inhibition of *TMEM16A* function blocks the slow wave (Hwang et al., 2009); and (d) knockout of the *TMEM16A* gene leads to the disappearance of the slow wave in intestinal smooth muscle (Hwang et al., 2009).

Current studies have found that diarrhea induced by pathogenic bacteria is associated with the excessive activation of chloride channels. Enterotoxins can elevate intracellular cyclic nucleotide (cAMP and cGMP) and Ca^2+^ levels, leading to CFTR and CaCC activation (Akabas, 2000). Elevated Ca^2+^ levels also promote the formation and release of arachidonic acid, prostaglandin E2 (PGE2), and 5-hydroxytryptamine (5-HT). Water and electrolytes are shifted to the outside of the enterocytes under the influence of these compounds (Peterson and Whipp, 1995; Dubreuil, 2012). The antidiarrheal effects of three classes of chemical-molecular CFTR inhibitors, namely, CFTR_inh_-172, BPO-27, and GlyH-101, have been proven in mouse models of cholera and STa toxin-induced intestinal fluid secretion (Thiagarajah et al., 2004; de Hostos et al., 2011; Cil et al., 2017). In addition to infections by intestinal pathogenic bacteria, infections by viruses and parasites are also important causes of secretory diarrhea. Notably, rotavirus is a common cause of diarrheal diseases in children (Lundgren and Svensson, 2001). In rotaviral diarrhea, NSP4, a nonstructural protein secreted from rotavirus, can directly or indirectly trigger the endoplasmic reticulum to release Ca^2+^ (Tian et al., 1994; Ball et al., 1996; Lorrot and Vasseur, 2007). Some chemosynthetic and natural inhibitors against intestinal CaCC have been proven to be highly effective at reducing watery stools in neonatal mice infected with rotavirus (Ko et al., 2014; Tradtrantip et al., 2014; Jiang et al., 2016; Yu B et al., 2018), which suggests that CaCC is the main channel involved in the intestinal secretion caused by rotavirus infection and that treating rotavirus diarrhea with CaCC inhibitors is feasible.

In addition to the usefulness of oral rehydration salts and antibiotics, the discovery of secretion and motility inhibitors has received wide attention in the study of antidiarrheal therapy (Lappe et al., 2017). Our present study aimed to identify new Cl^–^ channel inhibitors and systematically investigate the underlying mechanisms of Cl^–^ secretion and motility in the intestine. Furthermore, we evaluated the antidiarrheal effects in mouse intestinal closed-loop models and CT-induced and rotavirus-infected mouse diarrhea models to determine the potential utility of Cl^–^ channel inhibitors for major diarrheal diseases.

## Materials And Methods

### Cell Lines, Animals, And Chemicals

Fischer rat thyroid (FRT) epithelial cells that were stably transfected with *TMEM16A* and halide sensor YFP-H148Q/I152L were provided by Prof. Tonghui Ma (College of Basic Medical Sciences, Dalian Medical University, Dalian, China) and cultured in Coon’s modified F12 medium (Sigma-Aldrich, USA). HT-29 cells (ATCC HTB-38) were grown in McCoy’s 5A medium (Sigma-Aldrich, USA). The media for the FRT and HT-29 cells were supplemented with 10% fetal bovine serum (HyClone, USA), 100 U/ml penicillin, 100 µg/ml streptomycin, and 2 mM L-glutamine (Sigma, USA). Cells were grown in a 5% CO_2_ incubator at 37°C.

C57 mice (8–10 weeks) were maintained under specific pathogen-free conditions and fed a normal chow diet at Dalian Medical University (Permit Number: SCXK Liao 2013-0003).

CFTR_inh_-172, amphotericin B, indomethacin, amiloride, forskolin (FSK), 3-isobutyl-1-methylxanthine (IBMX), cell-permeable cAMP (CPT-cAMP), clotrimazole, ouabain, T16A_inh_-A01, cholera toxin (CT), and *Escherichia coli* heat-stable enterotoxin (STa) were purchased from Sigma-Aldrich. CaCC_inh_-A01, E_act_, and 5-HT were purchased from Tocris Bioscience (UK). Nystain was purchased from Amresco Limited Liability Company (USA). Fluo-4 NW was purchased from Life Technologies Corporation (USA). Plumbagin, ATP, ionomycin, thapsigargin, carbachol, genistein, vasoactive intestinal peptide (VIP), and PGE2 were purchased from Meilun Biotech Co. Ltd. (China). SA-11 rotavirus was a gift from Prof. A.S. Verkman (Departments of Medicine and Physiology, University of California San Francisco, San Francisco, CA, USA).

### Iodide Influx Measurements

Fluorescence assays were performed as described previously (Namkung et al., 2011). Briefly, FRT cells expressing *TMEM16A* and YFP were plated into 96-well black-walled clear bottom microplates (Corning, USA) and incubated until cells reached confluence. After being washed three times with I^–^free PBS, cells were incubated with different concentrations of plumbagin for 20 min. The fluorescence of each well in the plate was measured with the FLUOstar Optima fluorescence plate reader (BMG Lab Technologies, Germany) equipped with syringe pumps and fixed excitation/emission (500 ± 10 nm/535 ± 15 nm) filters. PBS was set as the negative control, and 100 µM ATP was set as the positive control.

### MTT Assay

Cell viability was measured using the MTT assay. Briefly, *TMEM16A*-expressed FRT cells were seeded in a 96-well plate and incubated at 37°C and in 5% CO_2_ for 24 h. After that, cells were treated with different concentrations (5–400 µM) of plumbagin for 24 h and then treated with MTT solution (5 mg/ ml) at 37°C for 4 h. DMSO (150 µl) was added into the wells to dissolve the formazan after removing the original solution. The OD values were recorded at 495 nm using microplate reader (Thermo, China). The rate of cell viability was calculated by the following formula: (OD_plumbagin_/OD_DMSO_) × 100%.

### Short-Circuit Current Measurements

FRT cells stably expressing *TMEM16A* and HT-29 cells that were grown on Snapwell inserts (1.12-cm^2^ surface area) were mounted in using chambers (Physiological Instruments, USA). FRT cells were bathed in half-Cl^–^ solution (apical, containing in mM: 65 NaCl, 65 Na gluconate, 2.7 KCl, 1.5 KH_2_PO_4_, 0.5 MgCl_2_, 2 CaCl_2_, 10 Hepes, and 10 glucose, pH 7.4) and HCO_3_^–^ buffered solution (basolateral, containing in mM: 130 NaCl, 2.7 KCl, 1.5 KH_2_PO_4_, 0.5 MgCl_2_, 2 CaCl_2_, 10 Hepes, and 10 glucose, pH 7.4) for 30 min. Then, the basolateral membrane was permeabilized with 250 μg/ml amphotericin B. HT-29 cells were bathed in symmetrical HCO_3_^–^ buffered solutions (in mM: 119 NaCl, 0.6 KH_2_PO_4_, 2.4 K_2_HPO_4_, 1.2 MgCl_2_, 1.2 CaCl_2_, 21 NaHCO_3_, and 10 glucose, pH 7.4). The buffers were aerated with 95% O_2_/5% CO_2_ at 37°C. The short-circuit current was measured using a VCC MC6 multichannel voltage/current clamp and recorded using Acquire and Analyze 2.3 (World Precision Instruments, USA).

For measurements in the intestine, the fresh colons that were removed from sacrificed C57 mice were washed with ice-cold modified Krebs-bicarbonate solution (containing in mM: 120 NaCl, 5 KCl, 1 MgCl_2_, 1 CaCl_2_, 10 glucose, 5 Hepes, and 25 NaHCO_3_, pH 7.4). After muscularis removal, small fragments of mucosa-submucosa were mounted in a 0.3-cm^2^ tissue holder, placed in using chambers bathed with Krebs-bicarbonate solution, gassed with 95% O_2_/5% CO_2_ and kept at 37°C throughout the experiment. To prevent the influence of prostaglandins and the epithelial sodium channel (ENaC), indomethacin (10 µM) was added to both the mucosal and serosal sides, and amiloride (50 µM) was added to the mucosal side.

### Cytoplasmic Calcium Measurements

Intracellular Ca^2+^ concentrations were measured using the fluorescent Ca^2+^ indicator Fluo-4 NW (Invitrogen, USA) as previously described (Ko et al., 2014). Briefly, HT-29 cells cultured for 48 h in 96-well black-walled microplates were loaded with Fluo-4 NW at 37°C for 30 min after removal of growth medium. Plumbagin was added to the wells and incubated for another 10 min. The fluorescence intensity was recorded continuously for 5 s, and 25 s more after adding ATP, CCh, or ionomycin using a fluorescence plate reader equipped with syringe pumps and custom Fluo-4 NW excitation/emission filters (485/538 nm).

### Fluid Secretion In Mouse Intestinal Closed Loops

Mice were starved for 24 h and anesthetized with intraperitoneal sodium pentobarbital (40 mg/kg). After a small abdominal incision, four closed mid-jejunum loops (∼15 mm) were isolated with sutures. Loops were injected with 100 µl of saline, saline containing STa (0.1 µg) or CT (1 µg) without or with plumbagin (20 µM). The abdominal incision was closed with a suture, and mice were allowed to recover from anesthesia. In 4 h, the mice were sacrificed with an intraperitoneal overdose of sodium pentobarbital (100 mg/kg), and the intestinal loops were removed. Intestinal fluid secretion was quantified from the loop weight-to-length ratio.

### CT- and Rotavirus-Induced Diarrhea Experiments

Neonatal C57 mice (aged 3–4 days) were randomly divided into three groups of five mice each: normal group, diarrhea model group, and drug treatment group. For normal group, mice received saline by oral gavage. For diarrhea model group, mice were inoculated with 3 μg CT or 25 μl (1.2 × 10^7^ pfu/ ml) of simian SA-11 rotavirus by oral gavage, a modification of previously reported models (Ko et al., 2014). To evaluate the anticholeraic effect, the mice in drug treatment group received 0.4 μg plumbagin before and twice daily after oral CT administration. To evaluate the antirotaviral diarrhea effect, mice received plumbagin twice daily after rotavirus infection. Stool samples were collected by gentle palpation of the mouse abdomen, and the water content was quantified as described with minor modifications (Yu B et al., 2018). A 1.5-mm-thick polydimethylsiloxane slab with a 1-mm-diameter hole was fabricated to contain a 1.17-mm^3^ volume of stool. The cylindrical stool plug was expelled onto a piece of a cellulose acetate membrane and allowed to contact the membrane for 2 min in a humidified atmosphere. The wetted area was recorded and quantified by digital imaging. In histological experiments, the mid-ileum was isolated, rinsed in PBS and fixed at 3 days after rotavirus inoculation. Specimens were embedded in paraplast, and paraffin sections were stained with hematoxylin and eosin. In order to establish the standard curve of wetting area and liquid volume, different volumes of saline were dropped on cellulose acetate membrane and measured the wetted area after 2 min.

### Gastrointestinal Motility

Adult C57 mice (8–10 weeks) were starved for 24 h and then divided into four groups of four mice each, administered respectively with saline, E_act_, plumbagin, and E_act_ plus plumbagin. Fifteen minutes later, mice were orally administered a charcoal meal (0.2 ml of 10% activated charcoal suspended in 5% gum acacia) over a 30-min period. Afterward, mice were sacrificed, and the small intestines were isolated. The peristaltic index was calculated as the percentage of distance traveled by the charcoal relative to the total length of the small intestine. For gastrointestinal motility of neonatal mice, 12 mice were divided into three groups and respectively orally administered with saline, rotavirus, and rotavirus plus plumbagin for 2 days then gavaged with 30 μl of activated charcoal and sacrificed after 15 min.

### Intestinal Smooth Muscle Contraction

The ileum was removed from the anesthetized mouse and washed with ice-cold Krebs-bicarbonate solution to clear intestinal contents. The ileal segments (∼10 mm) were connected to a force transducer and equilibrated in Krebs-bicarbonate solution for 60 min with a resting force of 1 mN. The bathing solution was changed every 20 min. Tension was monitored continuously using the DMT 750TOBS, a tissue organ bath system and analyzed using 750TOBS software (DMT Danish Myo Technology A/S, Denmark).

### Statistical Analysis

Statistical analysis was performed with Prism 7 software (GraphPad Software Inc., CA). Student’s t-test and one-way or two-way ANOVA followed by Bonferroni’s *post hoc* test were used to compare values between treatment and control groups. The results are summarized as the mean ± SEs or are shown as representative traces. Data were considered statistically significant when p < 0.05.

## Results

### Inhibition of *TMEM16A* Cl ¯ Transport by Plumbagin

**Figure 1:**
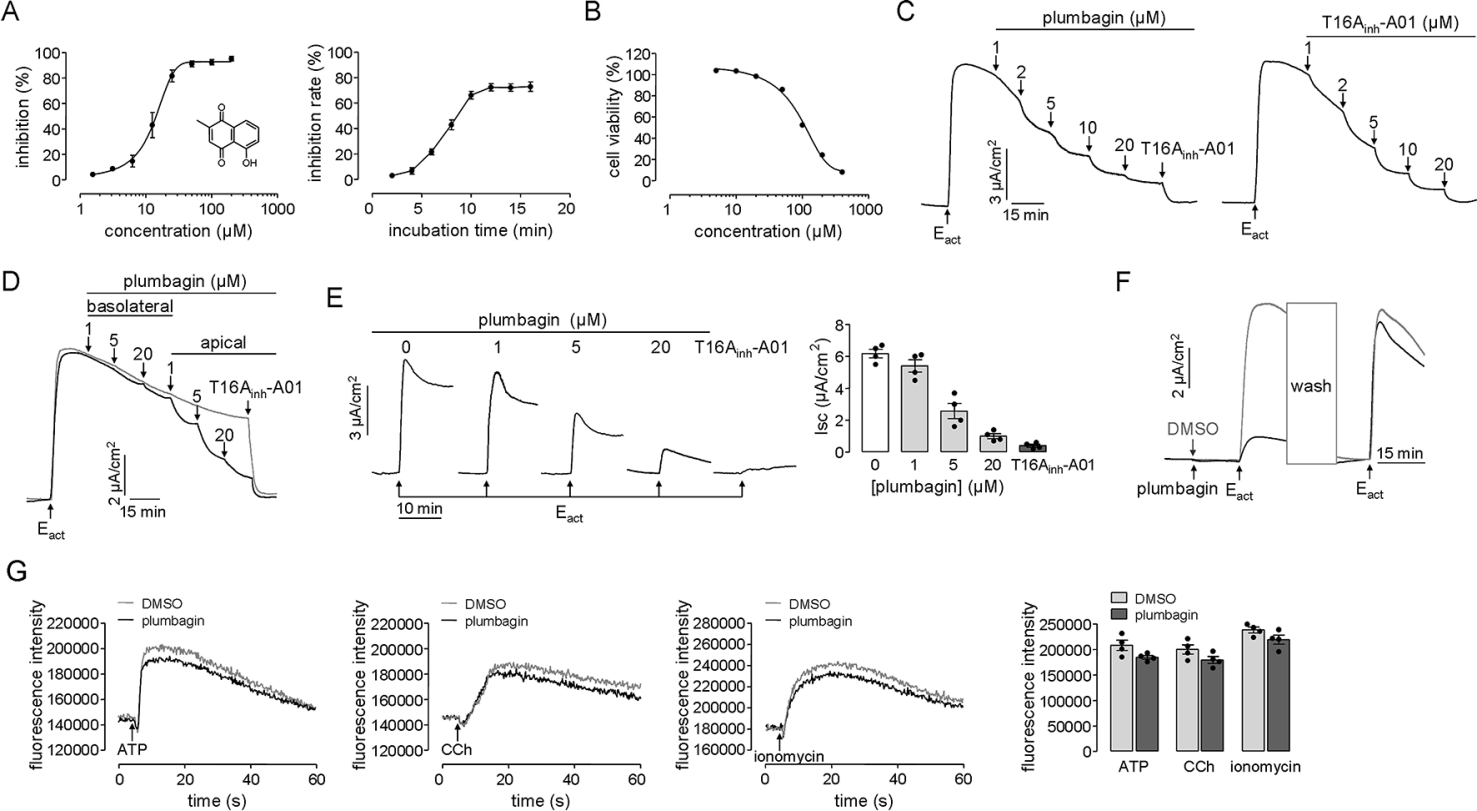
Inhibition of *TMEM16A* chloride channel activity by plumbagin. **(A)** Fluorescence assays in FRT-TMEM16A-YFP-H148Q/I152L cells. Left panel: Dose-dependent inhibition of *TMEM16A* activity by plumbagin and the structure of plumbagin. Right panel: Time-course inhibition of *TMEM16A* by plumbagin (n = 4). **(B)** MTT assay in *TMEM16A*-expressed FRT cells (n = 6). **(C)** Plumbagin inhibition of the short-circuit current induced by E_act_ (10 µM) in FRT cells expressing *TMEM16A*. T16A_inh_-A01 was used as a positive control. **(D)** Inhibition of the short-circuit current in FRT cells in which plumbagin was added first to the basolateral bathing solution and then to the apical bathing solution as indicated, following stimulation by 10 µM E_act_. **(E)** The blockage effect of plumbagin on *TMEM16A* activity. Left panel: Representative traces of short-circuit currents showing the inhibition of E_act_ (10 µM)-stimulated short-circuit current by 15 min of pretreatment with the indicated concentrations of plumbagin or with T16A_inh_-A01 (10 µM). Right panel: Dose-response summary (n = 4). **(F)** Reversibility of *TMEM16A* inhibition by plumbagin. After incubation with 20 µM plumbagin for 15 min, the cells were stimulated with 10 µM E_act_ and then washed, and the *TMEM16A*-mediated current was reactivated by E_act_. **(G)** Cytoplasmic calcium concentration measured by Fluo-4 NW fluorescence under basal conditions and following ATP (200 µM), CCh (100 µM), or ionomycin (1 µM) addition. HT-29 cells were pretreated with 20 µM plumbagin (n = 4).

The *TMEM16A* inhibitory effect of plumbagin was initially measured from the kinetics of I^–^ influx in *TMEM16A*-expressing FRT cells. As shown in **Figure 1A**, plumbagin inhibited the fluorescence quenching by the agonist ATP in a dose-dependent manner with an IC_50_ of approximately 12.46 μM. The inhibition of *TMEM16A* activity by 20 μM plumbagin reached its maximum after 10 min which seemed slower compared with T16A_inh_-A01 (Namkung et al., 2011). We assume that the effect might be related to its broad effect on cell viability. However, the result of cell viability assay showed that after 24 h treatment with 20 μM of plumbagin, the rate of FRT cell viability remained more than 90%. Therefore, the long onset time of plumbagin was not related to the influence of cell viability (**Figure 1B**).

The inhibition of *TMEM16A*-mediated chloride transport by plumbagin was further measured using short-circuit current assay. Plumbagin (1–20 μM) dose-dependently inhibited the E_act_-stimulated *TMEM16A*-mediated Clˉ current, in a manner that was similar to that of the *TMEM16A*-specific inhibitor T16A_inh_-A01 (**Figure 1C**). The results on the sidedness of plumbagin actions showed that basolateral addition of (1 and 5 μM) plumbagin had no inhibitory effect, while 20 μM plumbagin produced slight inhibition. However, inhibition by 1–20 μM plumbagin mainly occurred apically (**Figure 1D**). To investigate the blockage effect of plumbagin on *TMEM16A* activity, different concentrations of plumbagin (1, 5, and 20 μM) were first added to the apical side, and then, the short-circuit current was activated by E_act_. **Figure 1E** shows the dose-dependent inhibition of the *TMEM16A*-mediated Clˉ current by plumbagin, with a maximal inhibitory rate of ∼85% at 20 μM. Additionally, plumbagin inhibited *TMEM16A* chloride activity reversibly (**Figure 1F**). As shown in **Figure 1G**, 20 μM plumbagin slightly decreased ATP-, CCh-, and ionomycin-stimulated cytoplasmic Ca^2+^ concentration. The results indicated that plumbagin may have both inhibited and blocked activity of *TMEM16A* by directly interacting with extracellular sites and that this effect was reversible and independent of the intracellular calcium concentration.

### Inhibition of Intestinal Epithelial CaCC-Mediated Cl¯ Transport by Plumbagin

**Figure 2:**
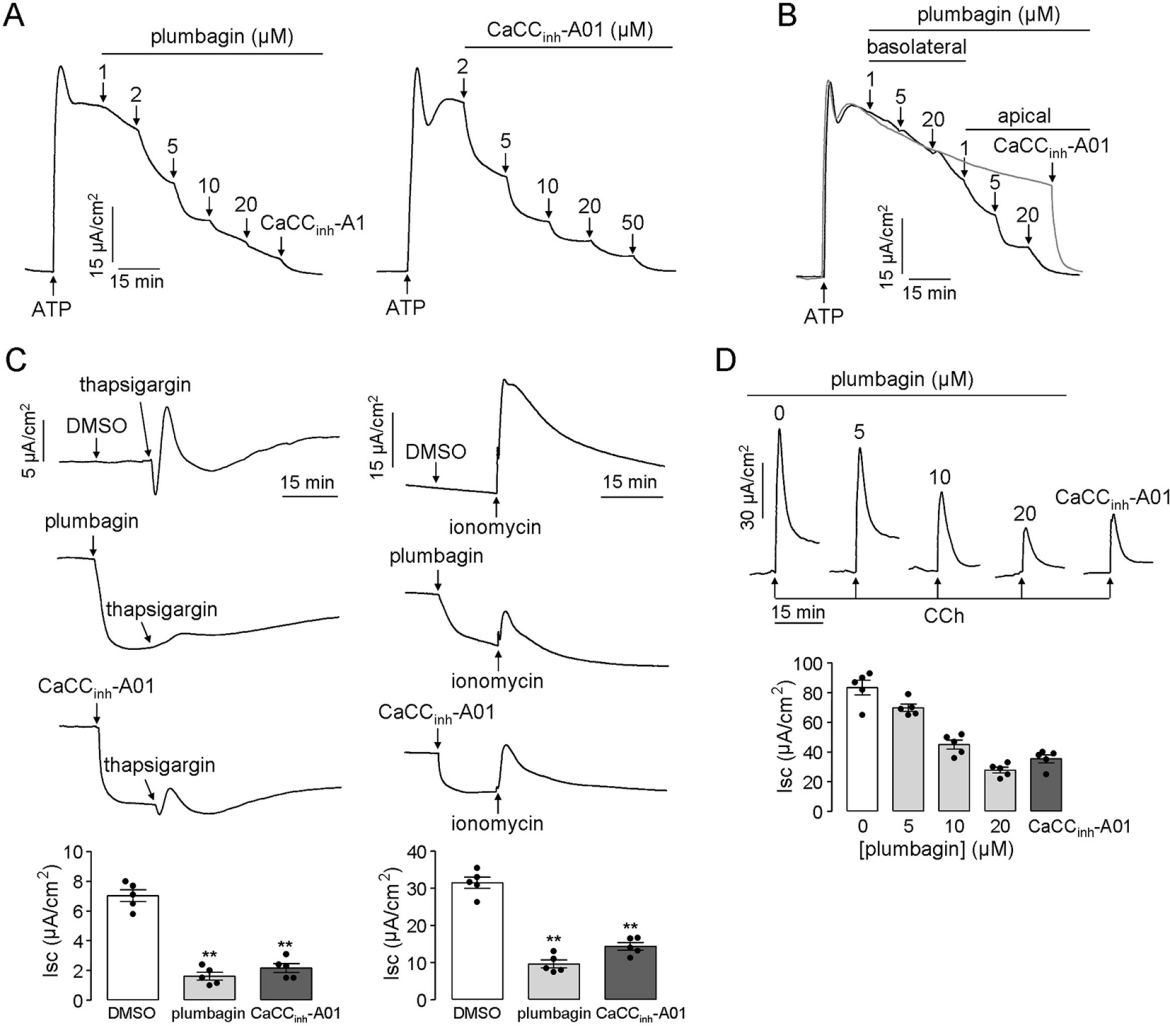
Plumbagin inhibits the CaCC current in HT-29 cells and mouse colons. **(A)** Inhibitory effects of plumbagin on the short-circuit current induced by ATP (200 µM) in HT-29 cells. CaCC_inh_-A01 was set as the positive control. **(B)** Inhibition of the short-circuit current in HT-29 cells in which plumbagin was added first to the basolateral bathing solution and then to the apical bathing solution as indicated, following stimulation with 200 µM ATP. **(C)** Short-circuit current in HT-29 cells showing inhibition of the thapsigargin (1 µM)- or ionomycin (1 µM)-stimulated short-circuit currents by 15 min pretreatment with plumbagin (20 µM) (n = 4). “**” indicate significantly different from the DMSO at the *P* < 0.01 level. **(D)** Representative traces showing inhibition of the CCh (500 µM)-stimulated short-circuit current in mouse colons by 20 min pretreatment with the indicated concentrations of plumbagin or CaCC_inh_-A01 (100 µM) (n = 5).

Some researchers pointed out that *TMEM16A* was a minor component of cacc conductance in airway and intestinal epithelial cells and indicated that there should be other types of CaCCs in epithelial cells (Namkung et al., 2011). Based on this speculation, we measured the inhibitory effect of plumbagin on intestinal epithelial CaCCs in human colonic carcinoma ht-29 cells that express endogenous CaCCs. **Figure 2A** shows that plumbagin (1–20 μM) inhibited the atp-induced short-circuit current in ht-29 cells. The remaining current was further inhibited by CaCC_inh_-A01 (50 μM), a type of CaCC-selective inhibitor. As shown in **Figure 2B**, basolateral addition of (1–5 μM) plumbagin had no inhibitory effect, whereas 20 μM plumbagin promoted slight inhibition. In contrast, the apical addition of 1–20 μM plumbagin produced the strongest inhibition of the current (**Figure 2B**). The inhibitory activity of plumbagin was also observed in short-circuit current assay in response to thapsigargin (calcium pump inhibitor) and ionomycin (calcium ionophore), which can increase intracellular calcium concentrations through different mechanisms. As shown in **Figure 2C**, 20 μM plumbagin inhibited most of the current produced by thapsigargin (1 μM) compared to dmso, and the efficiency was even better than that of CaCC_inh_-A01 (50 μM). A similar result was observed when the short-circuit current was stimulated by ionomycin (1 μM). Inhibition of the intestinal epithelial CaCC by plumbagin was further measured in freshly isolated mouse colonic mucosa-submucosa. Different concentrations of plumbagin were added into the mucosal solution 20 min before the addition of the cholinergic agonist cch. **Figure 2D** shows the inhibition of the CaCC current by plumbagin, with ∼12.1%, 46.3%, and 65.8% inhibitory rates for 5, 10, and 20 μM plumbagin, respectively. Additionally, the maximal inhibitory rate produced by plumbagin was slightly higher than that produced by CaCC_inh_-A01 (∼58.5%). these observations provided the basis for our conclusion that plumbagin could inhibit calcium-activated chloride secretion in the intestinal epithelium.

### Inhibition of CFTR Cl ¯ Transport by Plumbagin

**Figure 3:**
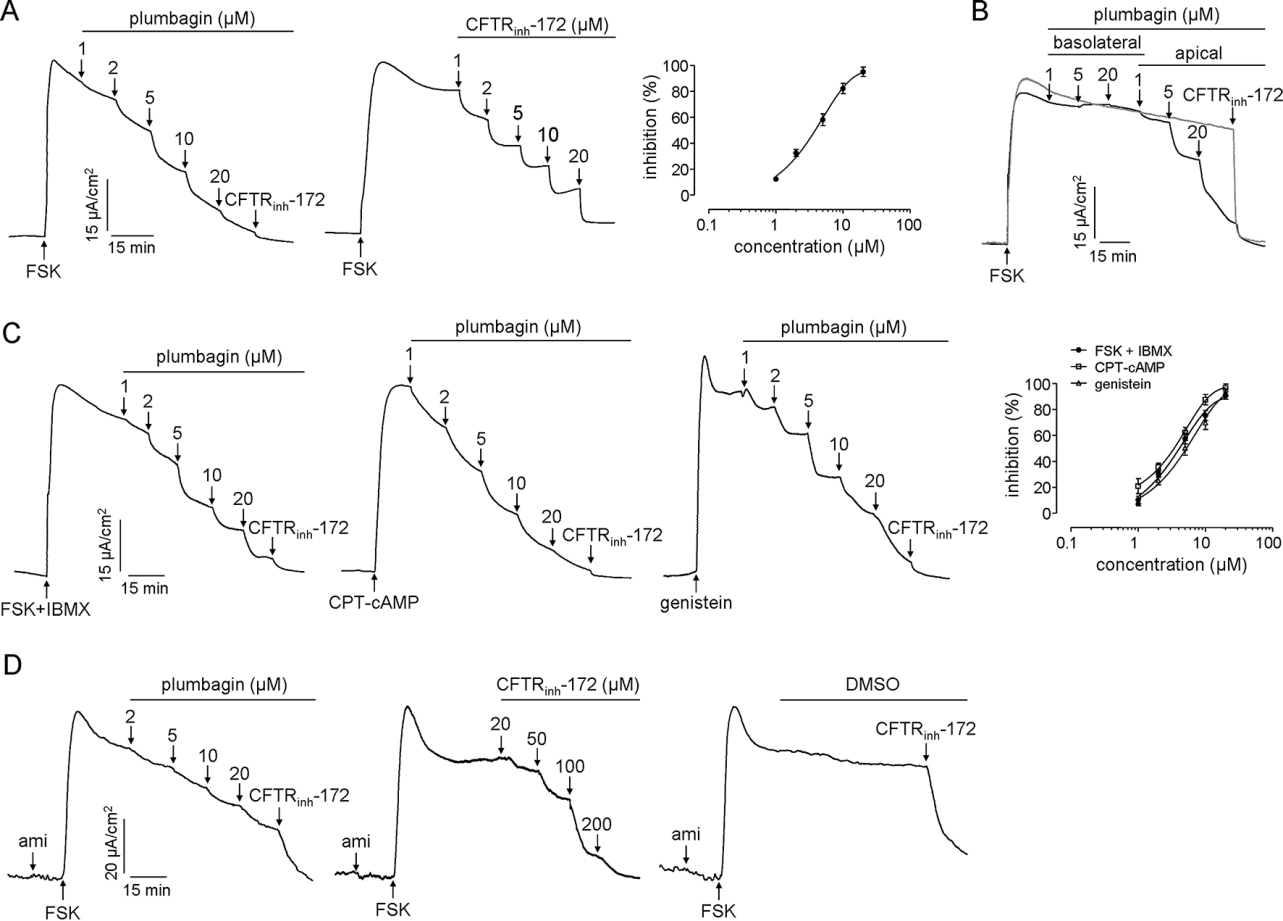
Inhibitory effects of plumbagin on CFTR Cl ¯ channel activity. **(A)** Short-circuit measurements showing plumbagin and CFTR_inh_-172 inhibition of the CFTR Cl ¯ current in HT-29 cells following stimulation by FSK (10 µM) (n = 3). **(B)** Inhibition of the short-circuit current in HT-29 cells in which plumbagin was added first to the basolateral bathing solution and then to the apical bathing solution, as indicated, following stimulation by 10 µM FSK. **(C)** Effects of plumbagin on the CFTR Cl ¯ currents induced by different agonists. Plumbagin was added to the apical side following CFTR activation by FSK (10 µM) plus IBMX (100 µM), CPT-cAMP (100 µM), or genistein (50 µM). CFTR_inh_-172 (20 µM) was added to the apical bathing solution to inhibit the remaining the CFTR current (n = 3). **(D)** Effects of plumbagin on CFTR Cl ¯ current in mouse colon. The indicated concentrations of plumbagin and CFTR_inh_-172 were added to the mucosal surface. Representative current traces are shown as one of three independent experiments.

CFTR in enterocytes provides the principal route for Cl^−^ secretion. Inhibition of CFTR activity significantly reduces intestinal fluid secretion. Therefore, the efficacy of plumbagin on CFTR chloride channel activity was investigated in HT-29 cells and colonic mucosa in mice. As shown in **Figure 3A**, the apical application of plumbagin inhibited the CFTR-mediated Clˉ current induced by FSK (adenylate cyclase activator) with IC_50_ of 3.9 μM, and the efficiency was approximately equal to that of CFTR_inh_-172. Basolateral addition of plumbagin did not produce any inhibition, whereas most inhibition was produced by the apical addition of plumbagin (**Figure 3B**). Additionally, IBMX (phosphodiesterase inhibitor), CPT-cAMP (nonhydrolysable cell-permeable cAMP analog), and genistein (direct CFTR activator) are all agonists that can activate CFTR in different mechanisms, so the inhibitory effects of plumbagin on short-circuit currents stimulated by these agonists were measured. The results showed that plumbagin could inhibit the FSK plus IBMX-, CPT-cAMP-, and genistein-stimulated currents dose-dependently in HT-29 cells with IC_50_ of 4.2, 3.6, and 5.5 μM (**Figure 3C**). These results indicated that the mechanism of CFTR inhibition by plumbagin was not associated with alterations in cAMP metabolism. Moreover, plumbagin also inhibited CFTR-mediated Clˉ current in the colonic mucosa-submucosa (**Figure 3D**).

### Effects of Plumbagin on the Activity of the Na^+^/K^+^ ATPase and K^+^ Channels

**Figure 4:**
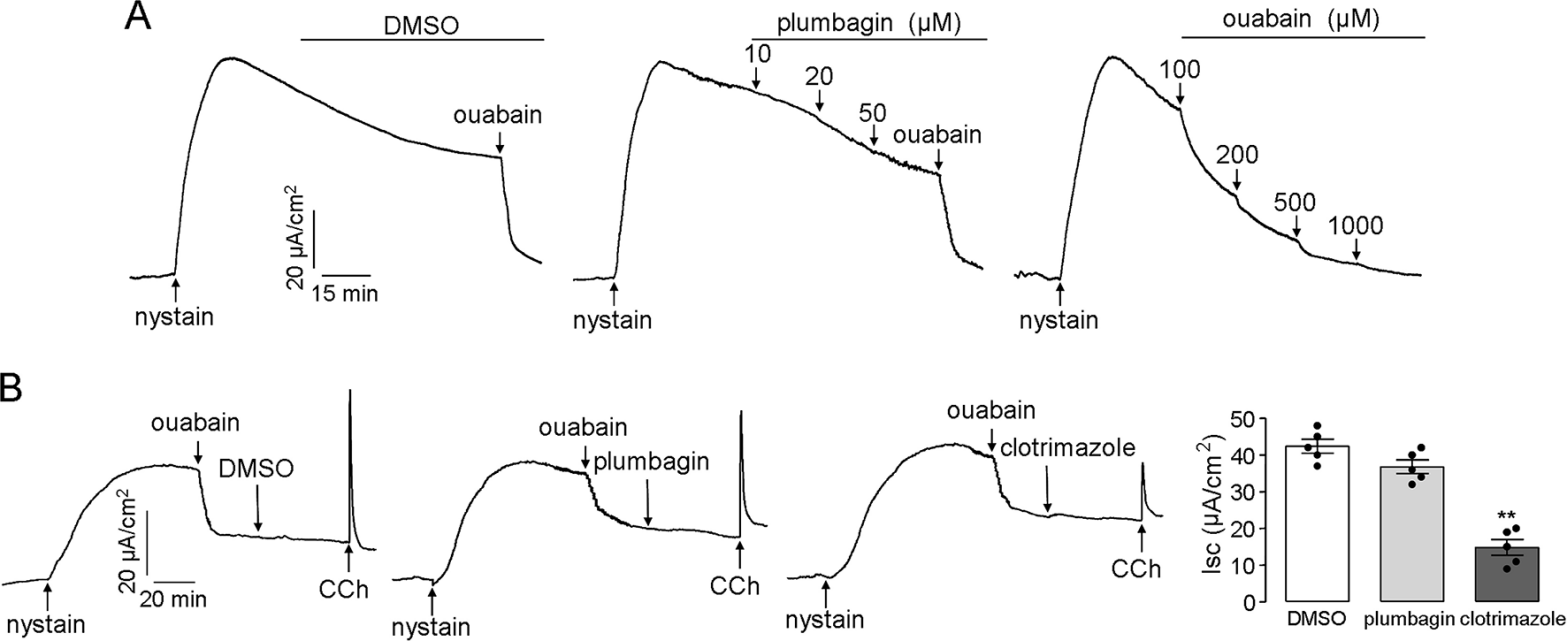
Effects of plumbagin on the activities of the Na^+^/K^+^ ATPase and K^+^ channels. **(A)** A short-circuit current corresponding to Na^+^/K^+^ ATPase was induced by the addition of nystain (200 mg/ml) into the mucosal solution, and the ouabain-sensitive current was set as an indicator of Na^+^/K^+^ ATPase activity. **(B)** Inhibition of Ca^2+^-activated K^+^ channels by DMSO, plumbagin (20 µM), and clotrimazole (50 µM) (n = 5). “**” indicate significantly different from the DMSO at the *P* < 0.01 level.

Na^+^/K^+^ ATPase and K^+^ channels that are located in the basolateral membrane provide the electrochemical driving force for chloride influx into enterocytes, and the transmembrane gradient of chloride can be abolished if the Na^+^/K^+^ ATPase and K^+^ channel activities are inhibited. Therefore, the effects of plumbagin on the Na^+^/K^+^ ATPase and K^+^ channels might be a mechanism that leads to the inhibitory activity of plumbagin on chloride transport; however, our results, shown in **Figure 4A**, refuted this assumption. After permeabilization of the mucosal membrane with nystain, the serosal application of plumbagin did not inhibit the Na^+^/K^+^ ATPase-mediated short-circuit current, which was completely abolished by ouabain. To measure the activity of plumbagin on K^+^ channels, after mucosal permeabilization with nystain, ouabain was first added to the serosal solution to inhibit Na^+^/K^+^ ATPase. Clotrimazole (a Ca^2+^-activated K^+^ channel inhibitor) was used as the positive control. As shown in **Figure 4B**, the serosal application of plumbagin had a slight inhibitory effect on the CCh-induced Ca^2+^-activated K^+^ channels. Plumbagin inhibition of Ca^2+^-activated K^+^ channels might be related to its activity in terms of [Ca^2+^] inhibition. These data demonstrated that the inhibitory effects of plumbagin on Clˉ channels were not connected to Na^+^/K^+^ ATPase and K^+^ channels and further suggested an extracellular site of action for CFTR and CaCC inhibition.

### Plumbagin Prevents Watery Diarrhea in Enterotoxin-Induced and Rotavirus-Infected Mice

**Figure 5:**
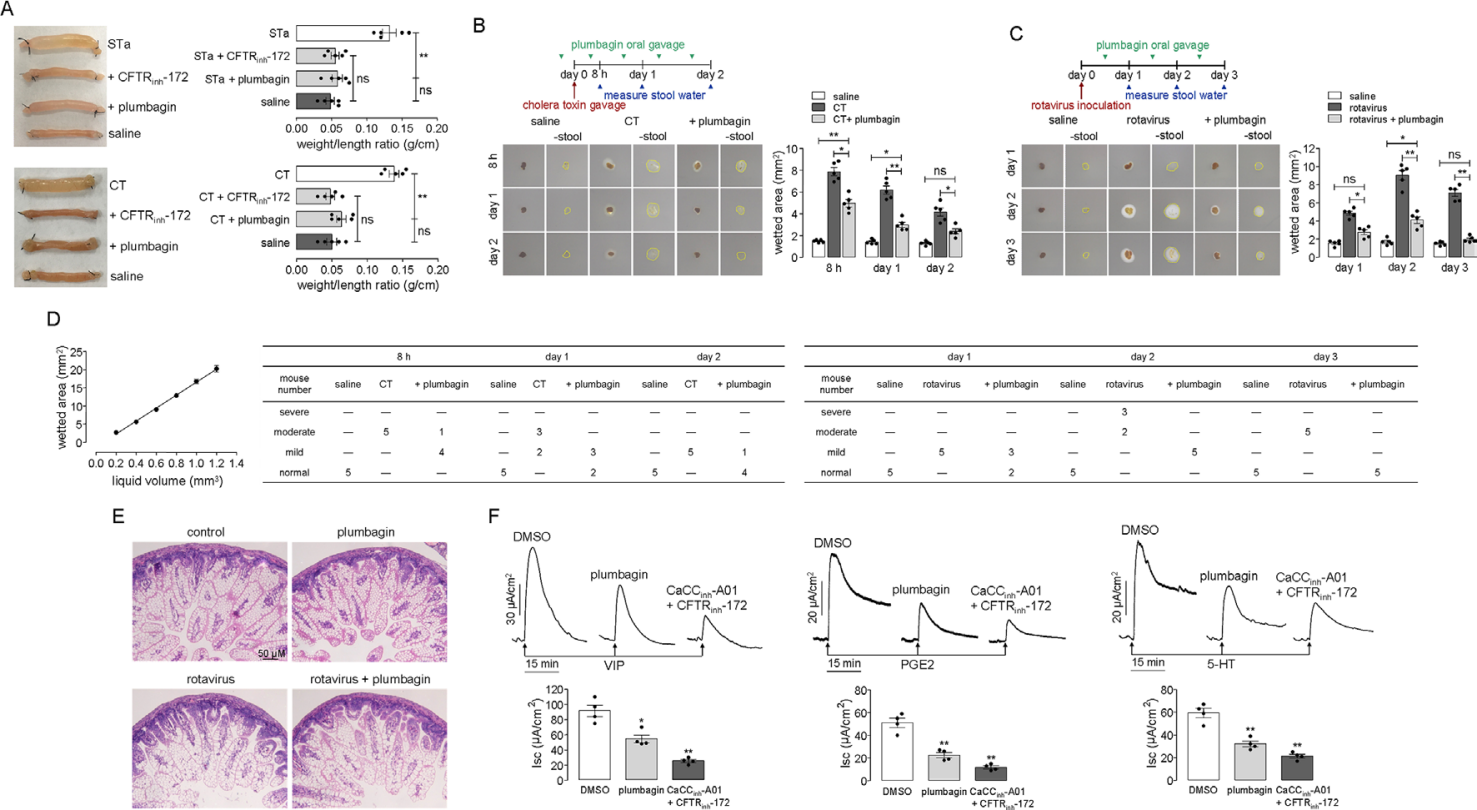
Antidiarrheal effect of plumbagin. **(A)** Photographs of isolated mouse ileal loops at 3 h after injection with 100 µl saline or saline containing STa (0.1 µg) or CT (1 µg) without or with plumbagin (20 µM). The bar graph shows the ileal loop weight/length (g/cm) ratio at 3 h (5 loops per group, **p < 0.01 and ns, no significant difference). **(B)** Top panel: Mice were infected with CT on day 0 and were orally administered plumbagin (0.4 µg) once on the day before the CT infection and twice per day thereafter. Middle panel: The wetted area is demarcated by a dashed yellow line. Bottom panel: Summary of the wetted area at indicated times (five mice per group, *p < 0.05, **p < 0.01). **(C)** Top panel: Mice were inoculated with rotavirus on day 0 and were orally administered plumbagin (0.4 µg) once per day thereafter. Bottom panel: Summary of the wetted area at indicated times (five mice per group, *p < 0.05 and **p < 0.01). **(D)** Standard curve relating wetted area to liquid volume (n = 4) and the number of mice with moderate, severe, mild diarrhea, or normal in each group. **(E)** Histology of the mouse ileum on day 3 after rotavirus inoculation. Hematoxylin- and eosin-stained paraffin sections from saline-control, plumbagin, rotavirus-inoculated, and plumbagin-treated mice (scale bar: 50 µm). **(F)** Inhibition of short-circuit currents induced by 100 nM VIP, 5 µM PGE2, or 10 µM 5-HT on the serosal side after pretreatment with DMSO, plumbagin (20 µM), or CaCC_inh_-A01 (100 µM) plus CFTR_inh_-172 (200 µM). Data summarized in the low panel are the means ± SEs from 4 replicates, *p < 0.05 and **p < 0.01.

The effect of plumbagin on fluid secretion was first tested in established models of STa- and CT-induced intestinal secretion in which fluid accumulation was tested in closed loops of the mouse mid-ileum. **Figure 5A** shows that the fluid accumulation in the STa- and CT-treated loops significantly increased compared to that in the saline-treated loops. The intraluminal application of plumbagin reduced both STa- and CT-induced intestinal fluid secretion.

To investigate the effect of plumbagin on diarrhea, plumbagin was tested in established mouse models of CT- and rotavirus-induced secretory diarrhea. The experimental design of the virus and CT infections, drug administration, and determination of fecal water content is shown in the top panels of **Figures 5B, C**. The middle panels of **Figures 5B, C** show images of the cellulose acetate membrane at 3 min after contacting a stool specimen, just before and after the physical removal of the stool mass. Mice receiving plumbagin had light-brown stool, similar to those of the normal mice, but the water content of their stools was significantly lower than that of the infected mice, especially on days 1 and 2 following CT infection and days 2 and 3 following rotavirus inoculation. The lower panels of **Figures 5B, C** summarize the wetted area data. According to the correspondence between saline volume and wetted area, we got the approximate linear equation: y = 19.193x − 1.7364 (r^2^ = 0.9892). If the stool volume was approximately considered 1.2 mm^3^, when the rate of liquid content was 100%, the wetted area was about 20.24 mm^3^; when the rate of liquid content was 50%, the liquid volume was 0.6 mm^3^, and the wetted area was about 9.02 mm^3^; when the rate of liquid content was 33%, the liquid volume was 0.4 mm^3^, and the wetted area was about 5.60 mm^3^; when the rate of liquid content was 16.6%, the liquid volume was 0.2 mm^3^, and the wetted area was about 2.68 mm^3^. According to the above relationship between liquid content and wetted area, the diarrhea severity in mice can be defined as follows: the rate of liquid content ≥50% was severe diarrhea, 33% ≤ rate of liquid content <50% was moderate diarrhea, 16.6% ≤ rate of liquid content <16.6% was mild diarrhea, rate of liquid content <16.6% was considered normal. **Figure 5D** shows the correlation between liquid volume and wetted area, and the severity of diarrhea in each group of mice.

Rotavirus infection of the intestine was then verified by histology (**Figure 5E**). On day 3 after rotavirus inoculation, large vacuoles were observed in the enterocytes lining most of the surface of the villi, with swelling of the villus tips, but this presentation was not observed in the enterocytes of the saline-treated normal mice. Similarly, there were no obvious pathological changes in the intestines of the mice after plumbagin treatment. The rotavirus-infected mice receiving plumbagin showed vacuolization that was similar to that in the untreated rotavirus-infected mice. Together, these data support the conclusion that plumbagin reduces the intestinal fluid secretion induced by enterotoxins on the luminal side and prevents watery diarrhea in enterotoxin-induced and rotavirus-infected mice while not affecting the rotavirus infection.

Elevations in VIP and the activation of its receptor are the main causes of tumor-associated and HIV-induced diarrhea (Manfredi et al., 1994; Reindl et al., 2004). Various invasive bacteria can induce the production of PGHS-2 and its products, such as PGE2, resulting in chloride secretion (Eckmann et al., 1997). The elevation in the 5-HT concentration is one of the pathological characteristics of IBS-D, and this elevation maybe the major cause of intestinal fluid secretion and motility enhancement (Spiller, 2011; Katsumata et al., 2017). Therefore, we measured the effects of plumbagin on VIP, PGE2, and 5-HT in mouse colonic epithelia. In this experiment, short-circuit currents were induced by VIP, PGE2, and 5-HT serosally after pretreatments with DMSO, plumbagin, and CaCC_inh_-A01 plus CFTR_inh_-172. As shown in **Figure 5F**, plumbagin inhibited VIP, PGE2, and 5-HT-induced short-circuit currents by ∼40%, 52%, and 42%, respectively, compared to DMSO, which was similar to the result produced by CaCC_inh_-A01 plus CFTR_inh_-172. These data confirmed the role of intestinal epithelial chloride channels, including CFTR and CaCC, in VIP-, PGE2-, and 5-HT-stimulated fluid secretion.

### Inhibition of Intestinal Motility and Smooth Muscle Contraction by Plumbagin

**Figure 6:**
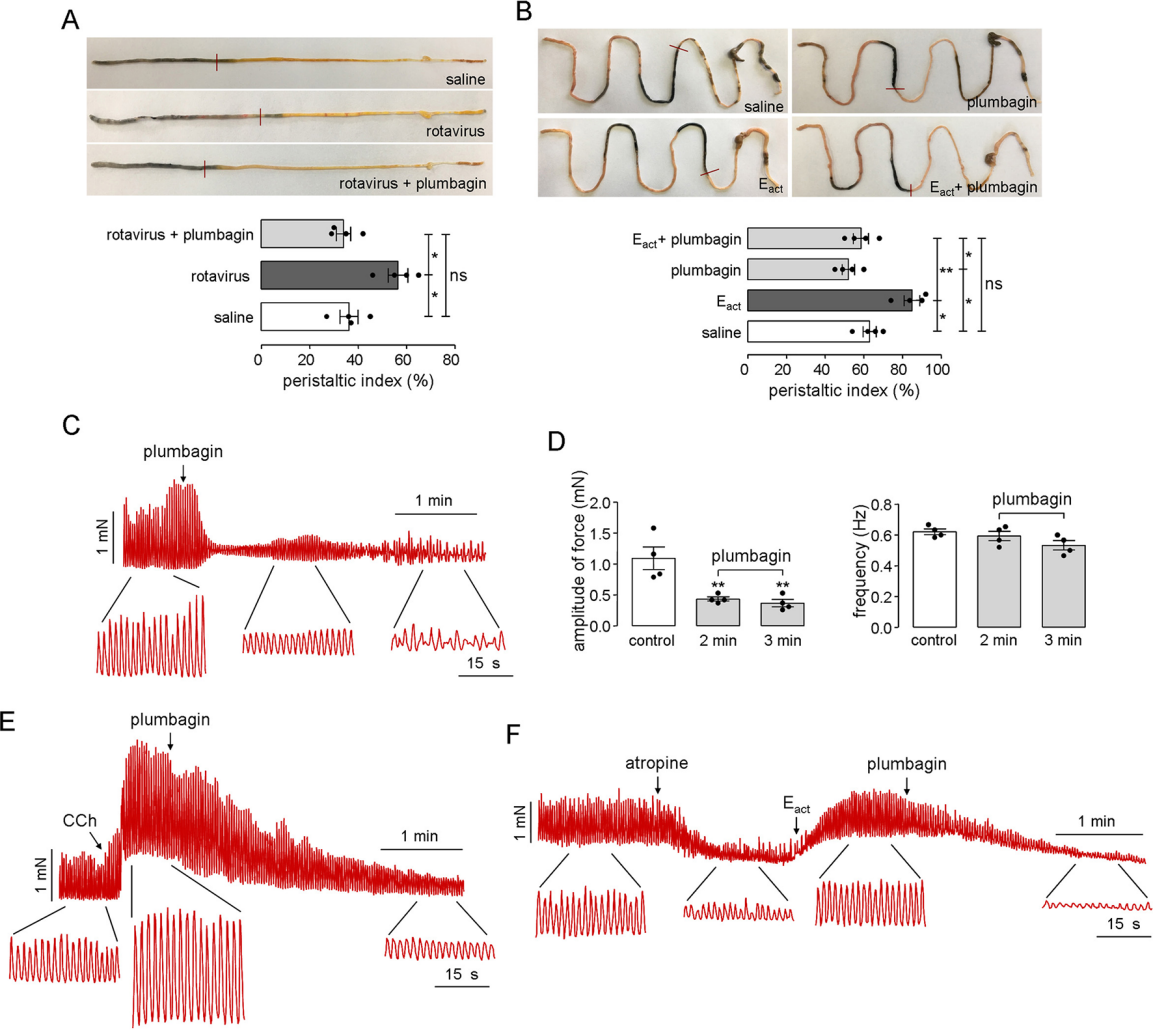
Inhibitory effects of plumbagin on intestinal motility and smooth muscle contraction. **(A)** Photographs of isolated neonatal mouse intestinal tracts showing the distances traveled by activated charcoal on day 2 after rotavirus infection and gavages of saline and plumbagin (20 µM). The bar graph shows the peristaltic indexes of activated carbon in the intestines (four mice per group, *p < 0.05). **(B)** Photographs of isolated mouse intestinal tracts showing the distances traveled by activated charcoal after the administration of saline and plumbagin (20 µM) by oral gavage and E_act_ (50 µM) by intraperitoneal injection. Peristaltic indexes of activated carbon in the intestines are shown in the bar graph (four mice per group, *p < 0.05, **p < 0.01 and ns, no significant difference). **(C)** Representative traces from mouse ileal segments showing the effects of plumbagin (20 µM) on basal contractions. **(D)** Plumbagin effect on the amplitude of force and contraction frequency (four ileal segments per group, **p < 0.01). **(E)** Representative traces from mouse ileal segments showing the effects of plumbagin (20 µM) on CCh (1 µM)-induced contractions. **(F)** Effects of plumbagin (20 µM) on smooth muscle contractions following atropine (1 µM) and E_act_ (10 µM) treatment. The representative current traces shown are from one of three independent experiments.

Intestinal motility disorder is one of the main pathophysiological features of diarrheas; therefore, the effects of plumbagin on intestinal motility were tested in both rotavirus-infected neonatal mice and adult mice. **Figure 6A** shows the distances traveled by the activated charcoal in the normal, rotavirus-infected, and plumbagin-treated neonatal mice. Rotavirus increased the peristaltic index in the neonatal mice on day 2 (55%) compared with that in the saline group (36%), which was decreased by plumbagin (34%), suggesting that the rotavirus infection resulted in increased intestinal motility while plumbagin could regulate intestinal motility close to the physiological level.

As shown in **Figure 6B**, after oral administration of plumbagin and activated charcoal, the peristaltic index in the adult mouse gut was 50.1%, which was lower than that of the saline group (66.2%). Because E_act_ could promote gastrointestinal wriggle via the activation of *TMEM16A*-mediated intestinal rhythm contractions, E_act_ was set as the positive control. After intraperitoneal injection of E_act_ (50 μM), the peristaltic index increased to 83.2%, and this effect was also inhibited by plumbagin, with a peristaltic index of 56.5%, which was close to the normal physiological level. The effect of plumbagin on mouse ileal smooth muscle contractions was further investigated in an *ex vivo* intestinal preparation. Contractile activity in isolated ileal segments from normal adult mice showed coordinated contractility and spontaneous rhythmicity, whereas significantly decreased contractility was observed at 2 min after treatment with plumbagin. Furthermore, the rhythmicity and coordination in the ileal segments were impaired at 3 min after plumbagin treatment (**Figure 6C**). Analysis of the tension recordings showed a significantly lower amplitude of force in the plumbagin-treated intestines than in the normal intestines, but there was no difference in the frequency of the contractions (**Figure 6D**). In addition to reducing the constitutive activity of the intestinal contractions, plumbagin also reduced the amplitude of the intestinal contractions following the application of CCh (**Figure 6E**). To determine whether the inhibitory effect of plumbagin on smooth muscle contractions was related to *TMEM16A*, atropine was first used to inhibit basal contractions. As shown in **Figure 6F**, E_act_ stimulated a large increase in contraction amplitude, and plumbagin inhibited the contraction amplitude to near 0 without affecting contraction frequency. Taken together, the results above suggested that plumbagin inhibited intestinal peristalsis through inhibition of *TMEM16A* activity in intestinal smooth muscle.

## Discussion

Secretory diarrhea is a health challenge in both developed and developing countries. The need for effective drug treatment for secretory diarrhea has not been met, particularly in developing countries, where cholera, viruses, and other enterotoxin-mediated secretory diarrheal diseases continue to be the leading cause of morbidity and mortality. Potential targets for diarrheal therapy include vaccines and antibiotics for pathogenic bacteria or viruses, enterocyte messengers (cAMP, cGMP, and Ca^2+^), ion channels (Cl^−^ and K^+^ channels), and transporters (NKCC1, NHE3, DRA, and SGLT1) (Venkatasubramanian et al., 2010; Das et al., 2018). Chloride channels, as the last step in Cl^−^ secretion, have become attractive targets for antisecretory therapy. Here, we demonstrate that plumbagin, an anthraquinone, inhibited the two major enterocyte Cl^−^ channels: CFTR and CaCC. In a previous study, by using FRT cells stably expressing human *TMEM16A* and a fluorescent protein (YFP-F46L/H148Q/I152L), plumbagin was show to function as a *TMEM16A* inhibitor (Seo et al., 2015). In this study, we determined the IC_50_ and time-course of plumbagin inhibiting *TMEM16A*. Although the onset time of plumbagin was relatively long, it was not related to the influence of cell viability which was consistent with the report by another research group (Subramaniya et al., 2011), so this inhibitory characteristic of plumbagin might be more likely due to its low affinity with *TMEM16A*.

Further, the underlying mechanism of *TMEM16A* inhibition by plumbagin as well as the effects of plumbagin on other chloride channels and cotransporters were studied in short-circuit current assay. The results indicated that plumbagin could directly and reversibly inhibit *TMEM16A*-mediated Cl^−^ currents on the apical side of cells in the concentration ranged from 1 to 20 µM, with no significant effect on calcium concentration. Since *TMEM16A* was identified, small molecules such as MONNA (IC_50_: 1.27 M), T16A_inh_-A01 (IC_50_: 1 M), dichlorophen (IC_50_: 5.49 M), and benzbromarone (IC_50_: 9.97μM) have been screened and identified as selective inhibitors of *TMEM16A* (Namkung et al., 2011; Huang et al., 2012; Oh et al., 2013). Remarkably, all of dichlorophen, benzbromarone, and plumbagin belong to the simple aromatic class, and their effective concentrations inhibiting *TMEM16A* were at micromole magnitude which seem a little high to apply directly *in vivo*. In addition to *TMEM16A* inhibitory activity, plumbagin was shown to be an enterocyte CaCC inhibitor based on evidence that plumbagin inhibited the currents induced by ATP and other calcium concentration-promoting reagents (thapsigargin and ionomycin). Regarding its CFTR inhibitory effect, plumbagin could effectively abrogate FSK-induced Cl^−^ secretion in HT-29 cells after FSK stimulation. Compared with the inhibition of *TMEM16A*-mediated currents by plumbagin, the inhibitory effect of plumbagin on CFTR was weaker and had longer onset time at 1 µM. Plumbagin could suppress CFTR currents larger than 95% at 20 µM; however, the inhibition rate of *TMEM16A* currents was less than 85% at the same concentration. It is speculated that the inhibitory activity of plumbagin on CFTR might be related to the effect of cAMP level. But in fact, plumbagin could effectively abrogate the Cl^−^ currents induced by CFTR agonists, including FSK plus IBMX, CPT-cAMP, and genistein at the same concentration it inhibited Cl^−^ currents induced by FSK, which indicated that the inhibitory effect of plumbagin on CFTR was not due to stimulation of phosphodiesterase. Additionally, plumbagin inhibited the activity of CaCC and CFTR on the apical side of cells and had no significant effect on the Na^+^/K^+^ ATPase and Ca^2+^-activated K^+^ channels in the basolateral membrane, so plumbagin most likely inhibited the activity of CaCC and CFTR by directly interacting with them.

Investigations that have contributed to a thorough understanding of the intestinal fluid transport mechanism have revealed the role of chloride channels in pathogen-induced secretory diarrhea. Some research studies have shown that heat-labile toxin (LT) secreted by *Enterotoxigenic Escherichia coli* (ETEC) and *Vibrio cholerae*, as well as cholera enterotoxin (CT) secreted by *Vibrio cholerae*, stimulate adenylate cyclase, resulting in an increase in the cAMP level and the activation of cAMP-dependent protein kinase A (PKA), ultimately leading to the phosphorylation of CFTR channels (Sears and Kaper, 1996; Vaandrager et al., 1997; Hug et al., 2003). ETEC also secretes two subtypes of heat-stable toxins (STs), namely, STa and STb (Rasheed et al., 1990; Arriaga et al., 1995). STa activates guanylate cyclase C (GC-C), which leads to cGMP accumulation and the phosphorylation of CFTR channels (Crane and Oh, 1997). STb binds to GTP-binding regulatory proteins, resulting in Ca^2+^ increases and the activation of calmodulin-dependent protein kinase II, which leads to the opening of the CFTR and CaCC channels (Saha and Bothast, 1999). CaCCs are also speculated to be the primary route for Cl^−^ secretion in diarrheas caused by rotavirus (Hempson et al., 2010), antiretroviral agents, and chemotherapeutic agents (Kahn et al., 2001; Rufo et al., 2004). In the present study, the antisecretory efficacy of plumbagin was investigated in mouse closed loops injected with either CT or STa, and the results indicated that plumbagin significantly inhibited both CT- and STa-induced fluid secretion. In antidiarrhea studies, watery stools induced by CT could be significantly inhibited by the oral gavage of plumbagin, proving that plumbagin might be useful in the treatment of diarrhea due to CFTR overactivation, including in cholera and Traveler’s diarrhea. In an antirotaviral diarrhea study, the oral gavage of plumbagin significantly reduced the water content of neonatal mice stools and alleviated diarrhea symptoms without an inhibitory effect on rotavirus infection. In previous work, we found that resveratrol oligomers *trans-ε-viniferin* and *r-2-viniferin* prevented rotaviral diarrhea in neonatal mice by inhibiting enterocyte CaCC activity (Yu B et al., 2018). Additionally, we also found that a naphthoquinone, namely, shikonin, decreased fluid secretion and delayed intestinal motility through the inhibition of intestinal epithelial CaCC and *TMEM16A* chloride channel activity, thus leading to inhibition of rotaviral diarrhea in mice (Jiang et al., 2016). Although the cellular details of CaCC remain to be fully elucidated, it is believed that CaCC chloride channel inhibitors are effective antirotaviral diarrhea drugs. In addition to carrying out their effects via a direct activation mechanism, pathogens can also increase the levels of neurotransmitters, neuropeptides, and hormones (5-HT, VIP, galanin, and prostaglandin) in the body (Matkowskyj et al., 2009) and indirectly activate Cl^−^ secretion (Wapnir and Teichberg, 2002). In our study, plumbagin exhibited evident inhibitory effects on VIP-, PGE2-, and 5-HT-stimulated Cl^−^ currents. Furthermore, because of the crosstalk between cyclic nucleotide and calcium signaling in enterocytes (Hoque et al., 2010; Namkung et al., 2010), compounds that inhibit both CaCC and CFTR might be beneficial in multifactorial diarrheal diseases.

Regarding fluid secretion mechanisms, increased peristalsis is another important cause of fluid loss in secretory diarrhea. The ICC participate in electrical activity transmission as a pacemaker for rhythmic electrical activity and constitute a bridge between nerves and smooth muscle (Sanders and Ward, 2006). Dysfunctions in the ICC lead to a variety of gastrointestinal motility disorders (Isozaki et al., 1997; He et al., 2001; Bettolli et al., 2008). Recent studies have found that *TMEM16A* (also known as Dog1) is highly expressed in different sources of ICC and that the intestinal ICC network in *TMEM16A* gene knockout mice develops normally; however, the slow-wave activity of small intestinal smooth muscle disappears (Isozaki et al., 1997; He et al., 2001; Hwang et al., 2009). Klein et al. provide precise evidence that intestinal ICC not only modulate intestinal smooth muscle contractions by producing rhythmic slow-wave potential but also play an important role in the signal transduction between intestinal neurons and gastrointestinal smooth muscle (Klein et al., 2013). These results suggest that the Ca^2+^-activated chlorine channel *TMEM16A* may play a critical role in the regulation of intestinal motility. In our research, the intestinal motility of neonatal mice was significantly enhanced by rotavirus infection. Plumbagin inhibited intestinal smooth muscle contractions in terms of amplitude but had no significant effects on frequency. After prolonged action, plumbagin further inhibited the rhythm of the intestinal contraction; the appearance was nonrhythmic and less coordinated in the plumbagin-treated ileal segments, which was consistent with the appearance in *TMEM16A* gene knockout intestinal segments (Singh et al., 2014), suggesting that the inhibitory effect of plumbagin on intestinal peristalsis is related to the inhibitory activity of *TMEM16A*. Thus, *TMEM16A* inhibitors might be a new class of candidates for the development of intestinal motility suppressants.

Plumbagin is a main component of *Plumbago zeylanica* L. (Plumbaginaceae), which is widely distributed in Africa, the Middle East, Asia, and Europe (Mishra et al., 2013; Ito et al., 2018; Koutroumpa et al., 2018). Plumbagin has a variety of biological effects, including antibacterial, antioxidant, anticancer, antifungal, anti-inflammatory, neuroprotective, and hypolipidemic effects (Roy and Bharadvaja, 2018). Among these activities, the research on anticancer activity of plumbagin is more comprehensive. Currently, it is believed that NF-κB, STAT3, and Akt are main target genes regulated by plumbagin. In gastric cancer cells, plumbagin inhibited cell proliferation and promoted apoptosis through suppressing the NF-κB signaling (Li et al., 2012). In esophageal, pancreatic, breast, and lung cancer cells, plumbagin reduced cell viability, invasion, and migration through inhibiting the activity, phosphorylation, and signaling of STAT3 (Hafeez et al., 2012; Yan et al., 2013; Cao et al., 2018; Yu T et al., 2018). It was also reported that plumbagin mediated autophagic cell death, apoptosis, and antiproliferation by inhibiting PI3K/Akt/mTOR signaling pathway in multiple cancer cells (Kuo et al., 2006; Pan et al., 2015; Wu et al., 2016). Although no inhibition of intestinal epithelial channels and no antidiarrheal application of plumbagin has been reported, plumbagin has been shown to significantly reduce the severity of diarrhea and improve fecal consistency in rodent models of ulcerative colitis (Pile et al., 2013), which can be partially explained by the inhibitory effect of plumbagin on intestinal Cl^−^ secretion.

## Conclusion

Excessive fluid secretion and increased enterocinesia are typical characteristics in enterotoxin- and rotavirus-induced diarrhea. Our results demonstrated that plumbagin can be a potential candidate for antisecretory therapy for major and life-threatening diarrheal conditions. The antidiarrheal activity of plumbagin can be attributed mainly to its broad Cl^−^ channel inhibition, with proven activity and efficacy at the cellular level, both *in vitro* tissues and in mouse models, which not only adds a new biological effect to the list for this compound but also provides a new leading drug in the treatment of secretory diarrheal diseases, including cholera, rotavirus infections, and other diarrheal conditions associated with cAMP and/or Ca^2+^-activated Cl^−^ secretion and intestinal motility disorder. Furthermore, the low-cost, wide use, and minor side effects of plumbagin increase the possibility of clinical application in the treatment of secretory diarrhea, though further studies in humans are necessary.

## Data Availability Statement

All datasets generated for this study are included in the manuscript.

## Ethics Statement

All animals in this study were handled in accordance with the recommendations of the “Guide for the Care and Use of Laboratory Animals of the National Institutes of Health”, and the experimental protocol was approved by the Liaoning Normal University Committee on Animal Research. All surgical procedures were performed under sodium pentobarbital anesthesia to minimize suffering.

## Author Contributions

BY designed the project, performed the experiments, analyzed the data, and wrote the manuscript. XZ, XY, LJ and JX performed experiments and analyzed the data. TM discussed the data. HY designed the project, analyzed, discussed the data, and wrote the manuscript.

## Funding

This work was supported by the Natural Science Foundation Project of Liaoning Province (No. 2019-BS-155), the Scientiﬁc Research Fund of Liaoning Provincial Education Department (No. L201783646), the National Natural Science Foundation of China (Nos. 81973380 and 31471099), and the National Science and Technology Major Project in Major New Drug Discovery (No. 2017ZX09201-002-007).

## Conflict of Interest

The authors declare that the research was conducted in the absence of any commercial or financial relationships that could be construed as a potential conflict of interest.
